# Comparative Chloroplast Genomics, Codon Usage Bias, and Phylogenetic Placement of *Euonymus alatus*

**DOI:** 10.3390/cimb48080822

**Published:** 2026-08-12

**Authors:** Yuemei Zhao, Weiwang Kong, Yushan Huang, Rongxiang Zhang, Changjiang Qian

**Affiliations:** School of Biological Sciences, Guizhou Education University, Guiyang 550018, China; zhaoyuemei@gznc.edu.cn (Y.Z.); z2158599705@163.com (W.K.); 18386154686@163.com (Y.H.); rxzhang@gznc.edu.cn (R.Z.)

**Keywords:** *Euonymus alatus*, chloroplast genome, sequence alignment, codon bias, phylogeny

## Abstract

*Euonymus alatus* is a species of medicinal and ornamental value, yet high-quality chloroplast genome resources for this taxon remain scarce. The complete chloroplast genome of *E. alatus* was assembled and compared with 15 congeneric species to investigate genomic structure and evolutionary dynamics. The genome is 157,416 bp with a GC content of 37.3%, containing 131 genes and 156 repeats (67 tandem repeats, 49 dispersed repeats, and 40 SSRs). Divergence was concentrated in non-coding regions, with 14 hypervariable regions identified as potential markers. IR expansion occurred independently in *E. fortunei* and *E. japonicus*, while no genome-wide inversions were detected. 11 protein-coding genes were identified under positive selection, among which *clpP* exhibited the strongest signal, suggesting a possible role in adaptive evolution. Codon usage bias analysis revealed that both mutation pressure and natural selection shape codon usage patterns, with the latter playing a relatively prominent role; 17 optimal codons were identified. Phylogenetic analysis strongly supported *E. alatus* as sister to *E. phellomanus*. This study provides a valuable genomic resource for species authentication, phylogenetic revision, and breeding in this genus.

## 1. Introduction

Chloroplast genomes, as fundamental genetic systems of semi-autonomous organelles in plants and algae, are pivotal for understanding plant evolution, function, and biotechnological advancements [1]. Originating from a primary endosymbiotic event ~1.5 billion years ago [2,3,4], chloroplasts primarily function in photosynthesis [1]. The chloroplast genome is typically a circular, double-stranded DNA molecule (120–160 kb) with a quadripartite structure comprising LSC, SSC, and two IR regions [2,5]. Present in high copy numbers per cell, it exhibits a low mutation rate, roughly one-tenth that of the nuclear genome [6]. Its gene content (110–130 genes, including 70–80 protein-coding genes) is highly conserved across seed plants [7]. These features-conserved structure, low recombination, and maternal inheritance-make chloroplast genomes invaluable tools for phylogenetic reconstruction, particularly at familial and generic levels [8,9,10,11].

*Euonymus alatus* (Thunb.) Siebold, a deciduous shrub in the family *Celastraceae* [12], is native to East Asia and valued for its ornamental features [12,13,14]. It has also been used in traditional medicine for its anti-inflammatory and antioxidant properties [15,16,17,18,19,20]. As a representative of the taxonomically complex genus *Euonymus* (ca. 130 species) [14], reliable species delimitation and phylogenetic reconstruction within this group have long been challenging due to morphological convergence and limited traditional molecular markers [21,22]. Given the numerous advantages of chloroplast genome sequences in resolving phylogenetic questions, this resource offers a promising approach to addressing these challenges. Although over 70 *Euonymus* chloroplast genomes are now available in public databases, high-quality genomic resources for *E. alatus* in particular remain insufficient.

In this study, we assembled and annotated the chloroplast genome of *E. alatus* based on high-throughput sequencing data, and analyzed its genome features, codon usage, repeat sequences, and phylogenetic relationships. Our results provide a valuable genomic resource for species identification, interspecific evolutionary research, and phylogenetic reconstruction within the genus *Euonymus*.

## 2. Materials and Methods

### 2.1. Plant Materials, Library Construction, Cp Genome Sequencing, Assembly and Gene

#### Annotation

Fresh leaves of *E. alatus* were collected in Yangling, Shaanxi Province, China (107°59′28″ E, 34°17′18″ N) in May 2025 by Professor Y.M. Zhao. After collection, the leaves were quickly dried in silica gel for DNA extraction. Total genomic DNA was extracted from a single individual using a modified CTAB method [23]. Voucher specimens are deposited in the Guizhou Education University Herbarium under voucher number ZYM 25051 (GEUH). High-throughput sequencing was performed on the Illumina HiSeq X Ten platform by Biomarker Technologies Corporation (Beijing, China). Raw data were filtered using fastp v0.20.0 software [24] to obtain clean reads. The chloroplast genome was assembled using GetOrganelle v1.7.7.0 [25] with the following command: get_organelle_from_reads.py -1 EA_1.clean.fq -2 EA_2.clean.fq -o EA_output -R 10 -k 21,45,65,85 -F embplant_pt (EA denotes the sample abbreviation for *E. alatus*). The assembly result was annotated with Geseq v2.03 (https://chlorobox.mpimp-golm.mpg.de/geseq.html, accessed on 15 May 2026) [26]. Subsequently, MAFFT v7.520 software was used for alignment and correction with manual adjustments [27], and the annotation results were verified using Geneious Prime 2024.0.5 [28] by comparison with the reference chloroplast genome of *E. phellomanus* (GenBank accession: NC_057060). The chloroplast genome map of *E. alatus* was generated with OGDRAW [29], and the obtained sequence was submitted to NCBI (accession number: PZ405866). To assess the reliability of the assembled genome, we compared it with four existing *E. alatus* sequences from GenBank. Additionally, the chloroplast genome sequences of 15 *Euonymus* species were downloaded from NCBI, partially reannotated, and used for comparative analysis together with the *Euonymus* data from this study (Table S1).

### 2.2. Repeat Sequence and SSR Locus Analysis

The REPuter online program [30] was used to identify dispersed repeats in the chloroplast genome of *E. alatus*, including forward, reverse, complement, and palindromic repeats, with the following parameters: a Hamming distance of 3 bp, a maximum of 50 repeat calculations, and a minimum repeat length of 30 bp [31]. Tandem repeats were detected using the online program Tandem Repeats Finder (TRF) [32], with a minimum repeat length of 10 bp. MISA-web [33] was employed to analyze SSR motifs, with minimum repeat number parameters set to 10, 5, 4, 3, 3, and 3 for mono-, di-, tri-, tetra-, penta-, and hexanucleotide sequences, respectively.

### 2.3. Codon Usage Analysis

#### 2.3.1. Gene Sequence Processing

A total of 86 coding sequences (CDS) from the chloroplast genome of *Euonymus* were screened using the following criteria [34]: (1) removal of duplicate sequences; (2) presence of a start codon ATG and a stop codon (UAA, UAG, or UGA); (3) sequence length ≥ 300 bp. Finally, 53 CDS were retained for subsequent analysis; the excluded sequences are listed in Table S2. For codon usage bias analysis, stop codons and the codon UGG (encoding tryptophan, which has no synonymous variation) were excluded from the 64 codons of the standard genetic code, and RSCU values were calculated based on the remaining 60 sense codons.

#### 2.3.2. Synonymous Codon Usage Bias Analysis

CodonW software (available at https://sourceforge.net/projects/codonw/, accessed on 25 May 2026) was used to calculate indices including relative synonymous codon usage (RSCU), the effective number of codons (ENC), and the A, T, C, and G contents at the third codon position [35]. The CUSP program from the EMBOSS package (https://www.bioinformatics.nl/emboss-explorer/, accessed on 27 May 2026) was used to calculate the GC content at the first, second, and third positions (GC_1_, GC_2_, GC_3_) and the overall GC content (GC_all_). To obtain global estimates, all 53 CDSs were considered as a single dataset for calculating GC_1_, GC_2_, GC_3_, ENC, and other codon usage parameters. Correlations among these parameters were then analyzed using SPSS version 20.0 (IBM Corp., Armonk, NY, USA).

#### 2.3.3. Neutrality Plot Analysis

Regression analysis was performed with GC_12_ as the dependent variable and GC_3_ as the independent variable. A significant correlation suggests that codon usage bias is mainly influenced by mutation, whereas a non-significant correlation indicates that selection plays a greater role [36].

#### 2.3.4. ENC-Plot Analysis

A two-dimensional scatter plot was drawn with GC3 as the abscissa and the ENC value as the ordinate. The expected ENC curve was generated using the formula: ENC = 2 + GC_3_ + 29/[GC_3_^2^ + (1 − GC_3_)^2^] [37]. When the observed ENC of a gene was close to the expected ENC, the codon bias of that gene was considered to be affected only by mutation. When the observed ENC deviated substantially from the expected ENC, the codon bias was considered to be mainly influenced by natural selection. Additionally, factors influencing codon usage bias in the chloroplast genome were analyzed using the ENC ratio, calculated as (ENC_exp_ − ENC_obs_)/ENC_exp_ [38].

#### 2.3.5. PR2-Plot Analysis

With G_3_/(G_3_ + C_3_) as the abscissa and A_3_/(A_3_ + T_3_) as the ordinate, the direction and degree of base bias were determined by the vector emanating from the central point [39,40]. If mutation is the sole cause of codon usage bias, the usage frequencies of purines and pyrimidines are expected to be balanced. Conversely, if the usage frequencies of these two types of bases are unbalanced, it indicates that natural selection and other factors exert a greater influence on codon usage bias [34].

#### 2.3.6. Optimal Codon Analysis

First, codons with RSCU > 1 were screened as high-frequency codons. Second, following the classical assumption that genes with lower ENC values tend to be more highly expressed [41], the 53 CDSs were sorted by ENC value. The top 10% (5 genes) with the lowest ENC were tentatively designated as the putatively high-expression gene library, and the bottom 10% (5 genes) with the highest ENC as the putatively low-expression gene library. The RSCU values of the two libraries were calculated separately, and codons with ΔRSCU ≥ 0.08 (ΔRSCU = RSCU_high-expression_ − RSCU_low-expression_) were identified as candidate optimal codons. Finally, codons satisfying both conditions were designated as optimal codons.

### 2.4. Comparative Chloroplast Genome Analysis

#### 2.4.1. IR Contraction/Expansion Analysis, mVISTA Analysis, and Rearrangement Analysis

To detect possible IR contraction and expansion events, the IR/SC boundary positions of 16 Euonymus chloroplast genomes were analyzed. Chloroplast genome comparison was visualized using the Shuffle-LAGAN mode of mVISTA [42], and genome rearrangement analysis was performed using Mauve v1.1.3 [43].

#### 2.4.2. Sequence Divergence Analysis and Hypervariable Region Analysis

To assess sequence divergence among Euonymus species, the 16 genomes were aligned using MAFFT v7.520 [27]. DNASP v5.10 [44] was then used to calculate the number of variable sites in the complete chloroplast genomes, protein-coding regions, and non-coding regions. For sliding window analysis, gaps were treated as missing data, and the window length and step size were set to 600 bp and 200 bp, respectively [45].

### 2.5. Selection Pressure Analysis

For codon-based selection analyses, the 74 protein-coding genes were individually aligned using MAFFT v7.520 [27] and concatenated into a single supermatrix. A partition scheme allowed each gene or codon position to follow its own substitution model, with optimal models selected by ModelFinder [46] in IQ-TREE v2.0.7 [47]. Nodal support was assessed with 1000 ultrafast bootstrap replicates [48]. The resulting ML tree was used as input for codon-based selection analyses in HyPhy v2.5 [49]. Positive selection was screened across all 74 genes using FUBAR [50] (default 20 × 20 grid; ω, the ratio of nonsynonymous to synonymous substitution rates (dN/dS); sites with posterior probability ≥ 0.9 for ω > 1 considered selected). Genes with maximum posterior mean ω > 0.5 (*n* = 35) were then tested for episodic selection using MEME [51] at *p* < 0.05.

### 2.6. Phylogenetic Analysis of the Genus Euonymus

A phylogenetic tree was reconstructed based on 21 chloroplast genome sequences. All sequences were aligned using MAFFT v7.520 [27], and the resulting alignment was trimmed with trimAl v1.4.1 [52], using the -gt 0.8 parameter to remove gap-rich regions. Two methods were employed for tree reconstruction. Maximum Likelihood (ML) analysis was performed using IQ-TREE v2.0.7 [47], with the optimal nucleotide substitution model (GTR+F+G4) automatically selected by ModelFinder [46] based on the Bayesian Information Criterion (BIC), and branch support was assessed using 1000 ultrafast bootstrap replicates [48]. Bayesian Inference (BI) analysis was conducted using MrBayes v3.2.7a [53,54] under the same GTR+F+G4 model, running four Markov chains (one cold and three heated) for 500,000 generations with sampling every 500 generations, and discarding the first 25% of sampled trees as burn-in. Posterior probabilities (PP) were calculated to evaluate node support, and convergence was confirmed by checking the average standard deviation of split frequencies (<0.01) and effective sample sizes (ESS > 200). All resulting trees were visualized and edited in FigTree v1.4.4 [55] (available at https://github.com/rambaut/figtree/releases, accessed on 15 June 2026).

## 3. Results

### 3.1. Structural Characteristics of the Chloroplast Genome of E. alatus

**Figure 1:**
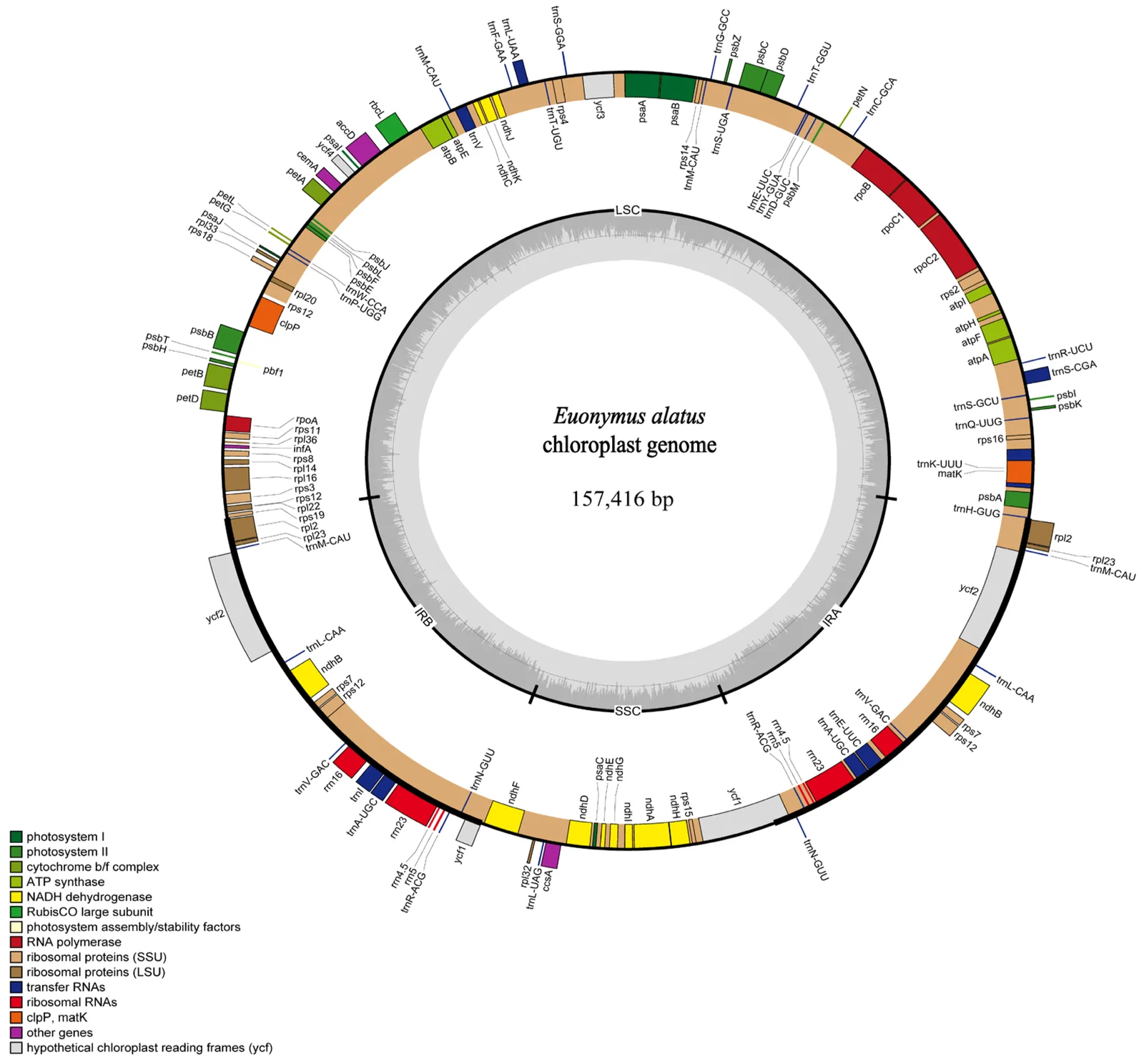
Physical map of *E. alatus* chloroplast genome.

A total of approximately 23 million paired-end raw reads were generated, yielding 22,836,344 clean reads (Q20 = 95.47%, Q30 = 88.06%) after quality filtering. The complete circular genome was assembled using GetOrganelle, and by mapping the clean reads back to the assembled genome, the average coverage was estimated at 387.5×; the two IR regions exhibited approximately twice the coverage of the LSC and SSC regions, consistent with the dual-copy nature of inverted repeats. The *E. alatus* plastome (157,416 bp) exhibited a typical quadripartite structure (Figure 1), consisting of LSC (86,172 bp), two IRs (26,354 bp each), and SSC (18,536 bp). IRscope boundary verification confirmed intact junctions (JLB: 86,172/86,173; JSB: 112,526/112,527; JSA: 131,062/131,063; JLA: 157,416/1), with the two IRs identical in length and sequence. The overall GC content was 37.3%, with GC contents of 35.2%, 31.7%, and 42.7% in the LSC, SSC, and IR regions, respectively.

To confirm the structural stability of the newly assembled genome, we performed whole-genome sequence alignment with four *E. alatus* chloroplast genomes retrieved from NCBI (accession numbers OP747447, OP581203, ON881435, and OK562424). During alignment, we found that the SSC region of OK562424 was in the opposite orientation relative to the other sequences, and its IRa region displayed a shifted position due to the choice of linearization start site. After correcting the SSC region by reverse complementation and adjusting the entire genome to the conventional linearization starting at the LSC/IRb (JLB) boundary, OK562424 achieved complete collinearity with the remaining sequences. These results indicate that the five chloroplast genomes of *E. alatus* are highly consistent in overall structure (the newly assembled genome shares >99% nucleotide identity with OP747447 and OP581203), further validating the structural stability and reliability of our newly assembled genome.

### 3.2. Analysis of Chloroplast Gene Types in E. alatus

Annotation of the *E. alatus* chloroplast genome revealed a total of 131 genes (Table 1), comprising 86 protein-coding gene copies (79 unique, with the remaining 7 duplicated in the IRs), 37 tRNA genes (7 duplicated in the IRs), and 8 rRNA genes (4 duplicated in the IRs). Seventeen genes contain introns (Table 2). Among them, *clpP* and *ycf3* each contain two introns, while *rps12* is a trans-spliced gene, with its 5′ end located in the LSC region and its 3′ end present as one copy in each IR region.

### 3.3. IR Expansion and Contraction Analysis

**Figure 2:**
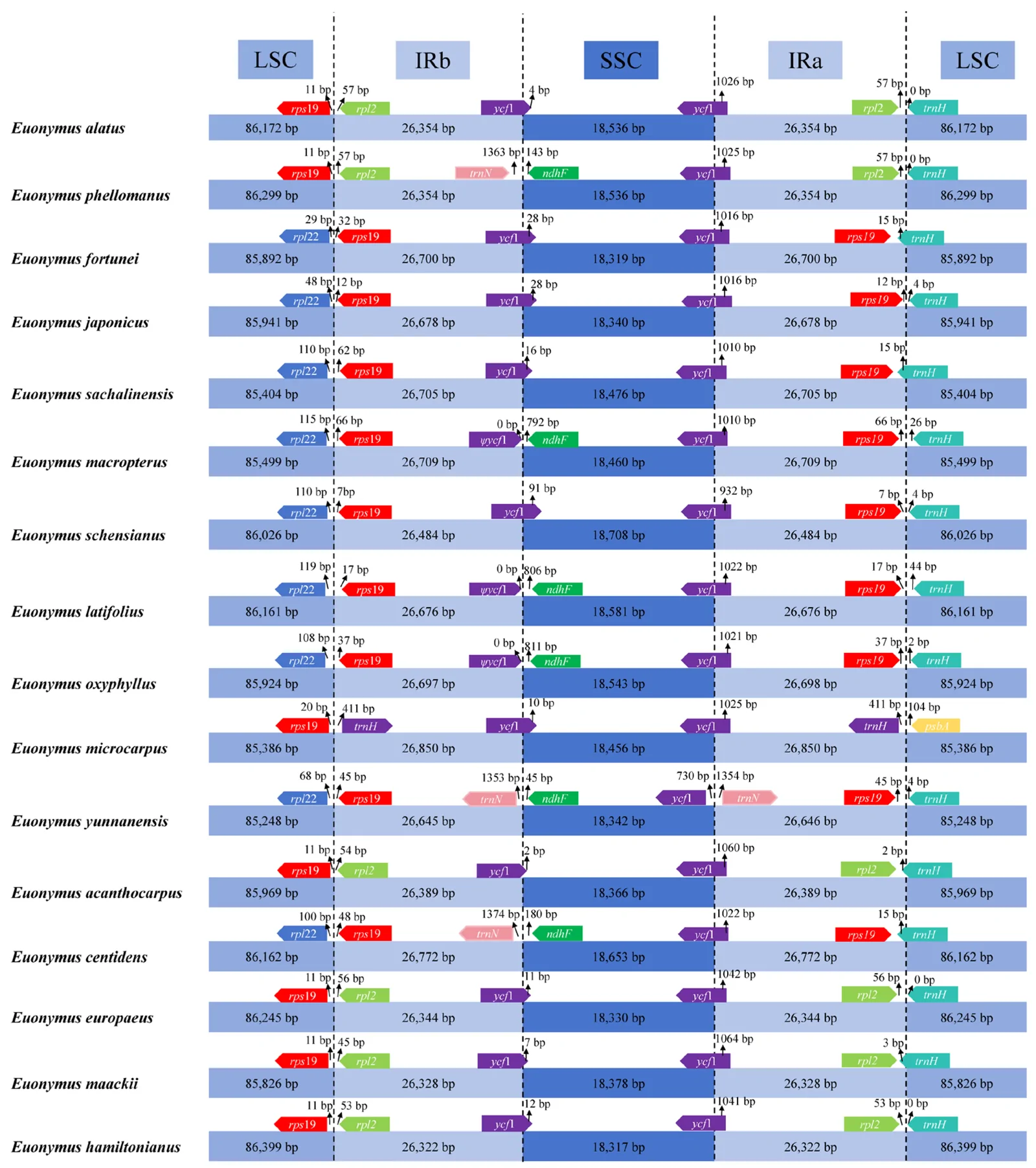
Analysis of IR/SC boundaries of 16 *Euonymus* chloroplast genomes.

Boundary analysis of the LSC, SSC, and IR regions in 16 *Euonymus* chloroplast genomes revealed the following (Figure 2). At the LSC/IRb junction, nine species shared boundary genes *rpl22* (LSC) and *rps19* (IRb), located 29–119 bp and 7–66 bp from the junction, respectively; six species had *rps19* (LSC) and *rpl2* (IRb), at 11 bp and 45–57 bp, respectively; and one species (*E. microcarpus*) had *rps19* and *trnH*, at 20 bp and 411 bp. At the IRb/SSC junction, ten species showed the junction within *ycf1*, with most of the gene in IRb and only 2–91 bp extending into SSC; three species had *trnN* and *ndhF*, located 1353–1374 bp and 45–180 bp from the junction; and three species had *ψycf1* and *ndhF*, directly at the junction and 792–811 bp away. At the SSC/IRa junction, 15 species had the junction within *ycf1*, with its IRa portion ranging from 932 to 1064 bp; one species (*E. yunnanensis*) had *ycf1* and *trnN* at 730 bp and 1354 bp. At the IRa/LSC junction, five species had the junction within *trnH* (IRa portion: 2–15 bp); four species had *rpl2* (IRa) and *trnH* (LSC) at 53–57 bp from the junction; six species had *rps19* (IRa) and *trnH* (LSC) at 7–66 bp and 0–44 bp; and one species (*E. microcarpus*) had *trnH* and *psbA* at 411 bp and 104 bp. Overall, the most notable boundary shifts occur at the IRb/SSC and SSC/IRa junctions, where *ycf1* is consistently truncated or duplicated across most species, identifying this gene as a hotspot for IR-mediated structural variation that may affect its functional integrity.

### 3.4. Comparative Analysis of Chloroplast Genomes

With *E. acanthocarpus* as the reference sequence, a global comparative analysis of 16 *Euonymus* species was performed using the mVISTA online software (https://webblast.ipk-gatersleben.de/misa/, accessed on 25 May 2026) (Figure S1). The results indicated high sequence similarity among the genes of the analyzed species, with variation levels in non-coding regions higher than those in coding regions. Accordingly, based on the nucleotide polymorphism results (Figure S2; Tables S3 and S4), the top seven most variable coding loci (*rpl22*, *ccsA*, *accD*, *rpl33*, *ndhF*, *rps15*, and *ycf1*; Pi > 4.9%) and non-coding loci (*psbI-trnS^GCU^*, *trnG^UCC^-trnR^UCU^*, *psbZ-trnG^GCC^*, *psbB-psbT*, *rps11-rpl36*, *rps19-rpl2* and *rpl2-trnH^GUG^*; Pi > 19%) were identified. Mauve analysis revealed no inversion rearrangements among these 16 species (Figure S3). Overall, the variation in the IR regions was lower than that in the LSC and SSC regions.

### 3.5. Analysis of Repetitive Sequences and SSRs in the E. alatus Chloroplast Genome

**Figure 3:**
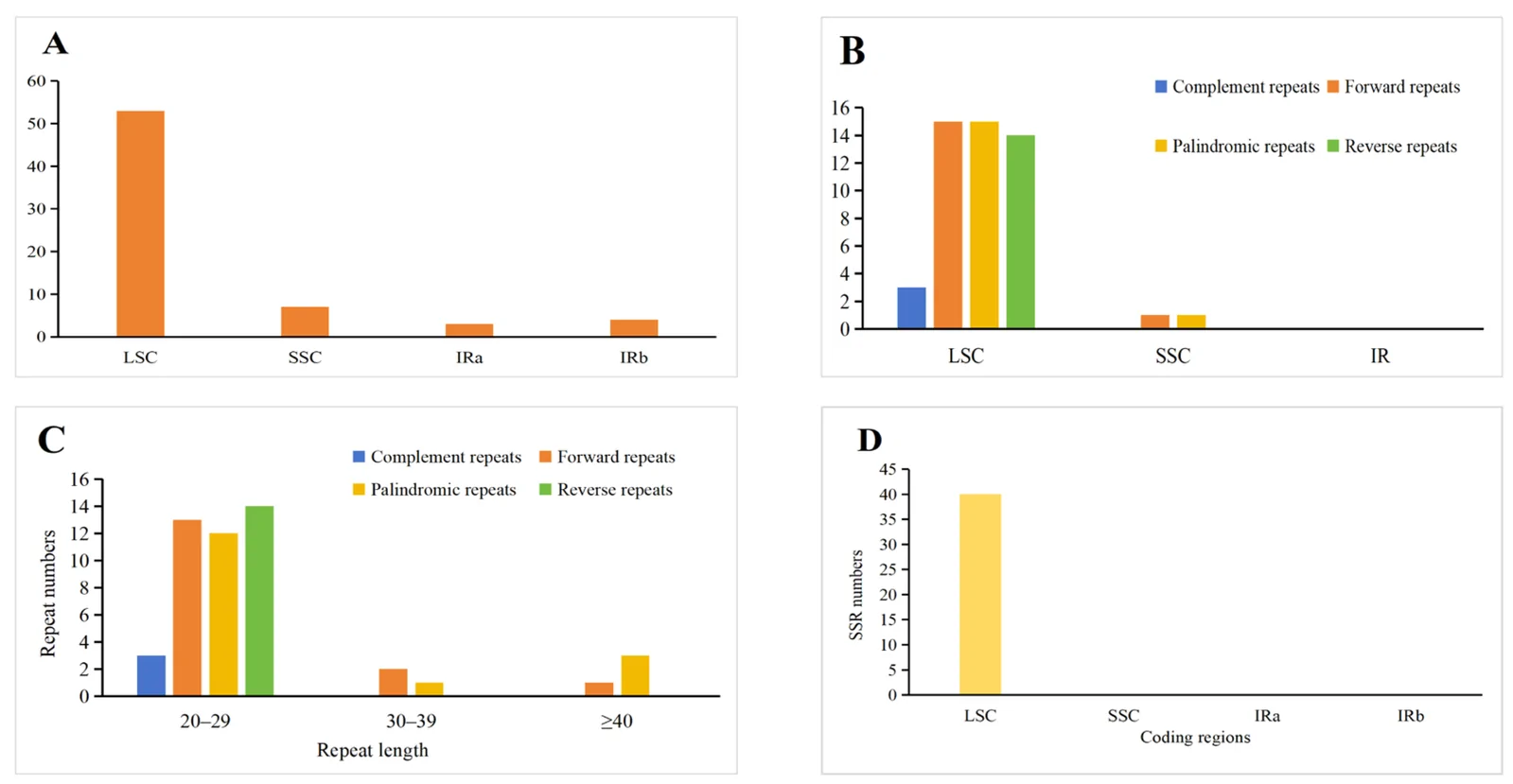
Number and types of repeats and SSRs of the *E. alatus* chloroplast genome. (**A**) Distribution of tandem repeats in LSC, SSC and IR regions. (**B**) Distribution of interspersed repeats in LSC, SSC and IR regions. (**C**) Distribution of interspersed repeats of different lengths. (**D**) Distribution of SSRs in different coding regions.

A total of 67 tandem repeats were detected in the *E. alatus* chloroplast genome, of which 53 were located in the LSC region, 7 in the IR region, and 7 in the SSC region (Figure 3A, Table S5). A total of 49 dispersed repeats were identified, including 16 forward repeats, 16 palindromic repeats, 14 reverse repeats, and 3 complementary repeats (Figure 3B,C; Table S6). Among these 49 repeats, the LSC region contained the highest proportion (95.9%), followed by the SSC region (4.1%), with no repeats located in the IR regions. Additionally, 40 SSR loci were detected, including 23 mononucleotides, 8 dinucleotides, 1 trinucleotide, 6 tetranucleotides, and 2 pentanucleotides (Figure 3D, Table S7). Mononucleotide repeats accounted for the highest proportion (57.5%) of all SSRs, and all 23 mononucleotide repeats were composed of A or T. Across all types of repeats (tandem repeats, dispersed repeats, and SSRs), the intergenic spacer (IGS) regions exhibited the highest abundance, while the coding sequences (CDS) regions exhibited the lowest abundance. Based on criteria of longer repeat units and IGS/intron locations, seven SSR loci (bold in Table S7) are recommended as priority candidates for molecular marker development, requiring further validation via primer design and polymorphism screening.

### 3.6. Analysis of Selection Pressure on the Chloroplast Genome

Using FUBAR and MEME, we investigated selective pressures on 74 chloroplast protein-coding genes from 16 *Euonymus* species. FUBAR analysis showed that the mean dN/dS (ω) values of all genes were far below 1 (overall mean 0.0256, range 0.0001–0.0787), and no codon sites were detected for pervasive positive selection (ω > 1 and posterior probability ≥ 0.9), indicating that these genes are predominantly under strong purifying selection (Table S8). To further detect episodic positive selection on specific branches, we performed MEME analysis on the 35 genes that exhibited maximum ω values > 0.5 in the FUBAR analysis and identified significant positive selection signals (*p* < 0.05) in 11 genes, involving a total of 97 codon sites (Table 3). Among them, *clpP* exhibited the strongest signal (82 sites), and other putatively positively selected genes included *psbM*, *petA*, *psaA*, *psbB*, *atpB*, *cemA*, *ndhA*, *ndhK*, *psbA*, *and rps19*, many of which are directly involved in photosynthesis, such as Photosystem II (*psbA*, *psbB*, *psbM*), Photosystem I (*psaA*), and the cytochrome b6f complex (*petA*).

### 3.7. Codon Usage Bias Analysis of the E. alatus Chloroplast Genome

#### 3.7.1. Synonymous Codon Usage Bias in the Chloroplast Genome of *E. alatus*

The average GC content of the 53 CDS in the chloroplast genome of *E. alatus* is 38.15% (Table S9). The GC_1_, GC_2_, and GC_3_ contents are 46.35%, 37.86%, and 30.26%, respectively. Except for three genes (*ndhC*, *cemA*, and *ycf2*) where GC_3_ content is higher than GC_2_, all other genes have GC_3_ content lower than GC_1_ and GC_2_, indicating that codons in this plant preferentially end with A or U. This A/U preference reflects mutational bias toward lower GC content and/or translational selection for codons recognized by abundant tRNAs. The ENC (effective number of codons) values of the 53 CDS range from 36.6 to 61.0, with an average of 48.31. Except for four genes (*rps14*, *rps18*, *rps8* and *rpl16*), all other genes have ENC values greater than 40, indicating that *E. alatus* exhibits an overall weak codon usage bias, a typical feature of chloroplast genomes under relaxed selective constraints.

**Figure 4:**
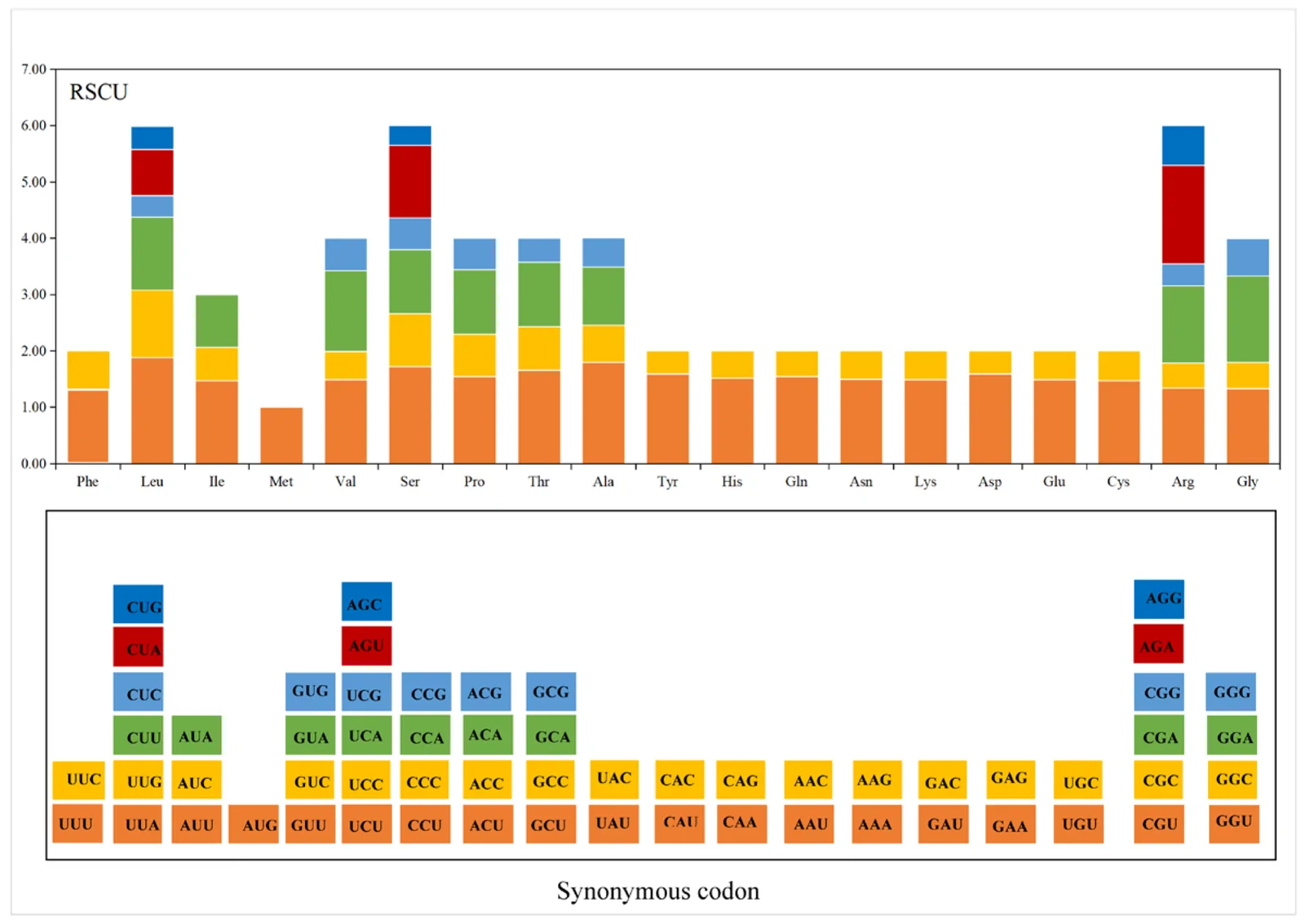
Summary of codon usage of the cp genome of *E. alatus*.

Among the codon usage parameters of the *E. alatus* chloroplast genome, GC_all_ shows highly significant correlations with GC_1_, GC_2_, and GC_3_. GC_1_ and GC_2_ also exhibit a highly significant correlation, and GC_1_ is significantly correlated with GC_3_; however, the correlation between GC_2_ and GC_3_ is not significant. These results indicate that GC_1_ and GC_2_ are similar in base composition, whereas GC_3_ differs considerably from them. Furthermore, all three GC components contribute to the overall GC content. ENC is significantly correlated only with GC_3_, suggesting that the base composition at the third codon position has a greater impact on codon usage bias (Table S10). This is because synonymous mutations at GC_3_ are largely neutral due to codon degeneracy, making GC_3_ the primary driver of codon usage bias, whereas GC_1_ and GC_2_ are more constrained by protein structure. Among the codons of *E. alatus*, 29 have RSCU > 1 (Table 4, Figure 4), of which 28 end with A or U, further indicating that this plant prefers codons ending with A/T, consistent with translational selection for codons matching abundant chloroplast tRNAs.

#### 3.7.2. Neutrality Plot Analysis Results

**Figure 5:**
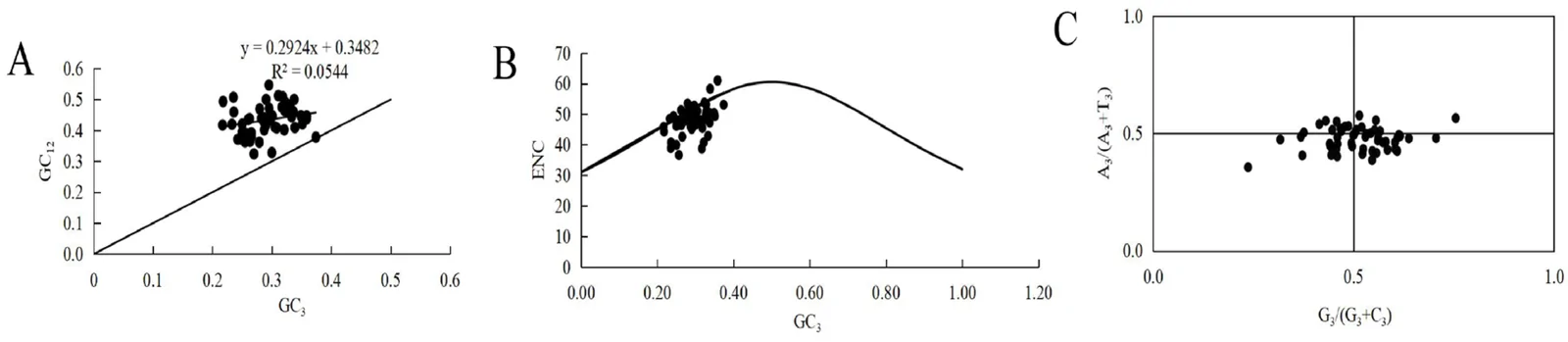
Neutrality plot analysis (**A**), ENC-plot analysis (**B**), and analysis of PR2 bias plot (**C**) of chloroplast genes of *E. alatus*.

Neutrality analysis (Figure 5A) showed no significant correlation between GC_12_ and GC_3_ (r = 0.233, *p* > 0.05), and the regression slope (0.2924) was considerably less than 1, suggesting that natural selection, rather than mutation pressure, dominates codon usage bias in the *E. alatus* chloroplast genome.

#### 3.7.3. ENC-Plot Analysis Results

The ENC values ranged from 36.6 to 61.0, with genes distributed both near and far from the standard curve (Figure 5B). Based on the ENC ratio (Table S11), 19 genes (36%) had ENC values between −0.05 and 0.05, indicating that codon usage in these genes is influenced by mutation pressure. The remaining 34 genes (64%) were affected by selection. These results suggest that although both mutation and natural selection influence codon usage in the chloroplast genome of *E. alatus*, natural selection plays a more significant role in shaping its codon usage bias. However, this analysis assumes codon usage is determined solely by mutation pressure; thus, the results are indicative and require validation by complementary methods.

#### 3.7.4. PR2-Plot Analysis Results

The PR2-plot analysis (Figure 5C) showed an uneven distribution of bases, with a bias towards G and T over C and A in the codon usage of the *E. alatus* chloroplast genome. This imbalance suggests that natural selection, rather than mutation pressure alone, plays an important role in influencing codon usage bias.

#### 3.7.5. Identification of Optimal Codons

There are 29 high-frequency codons (RSCU > 1) (Table 4) and 21 high-expression codons (ΔRSCU ≥ 0.08) (Table 5). The intersection of these two sets, comprising 17 codons, represents the optimal codons of the *E. alatus* chloroplast genome: UUU, UUA, UUG, CUU, AUU, GUA, CCU, ACU, GCU, GCA, UAU, CAU, AAA, GAA, UGU, CGA and GGA.

### 3.8. Phylogenetic Analysis

**Figure 6:**
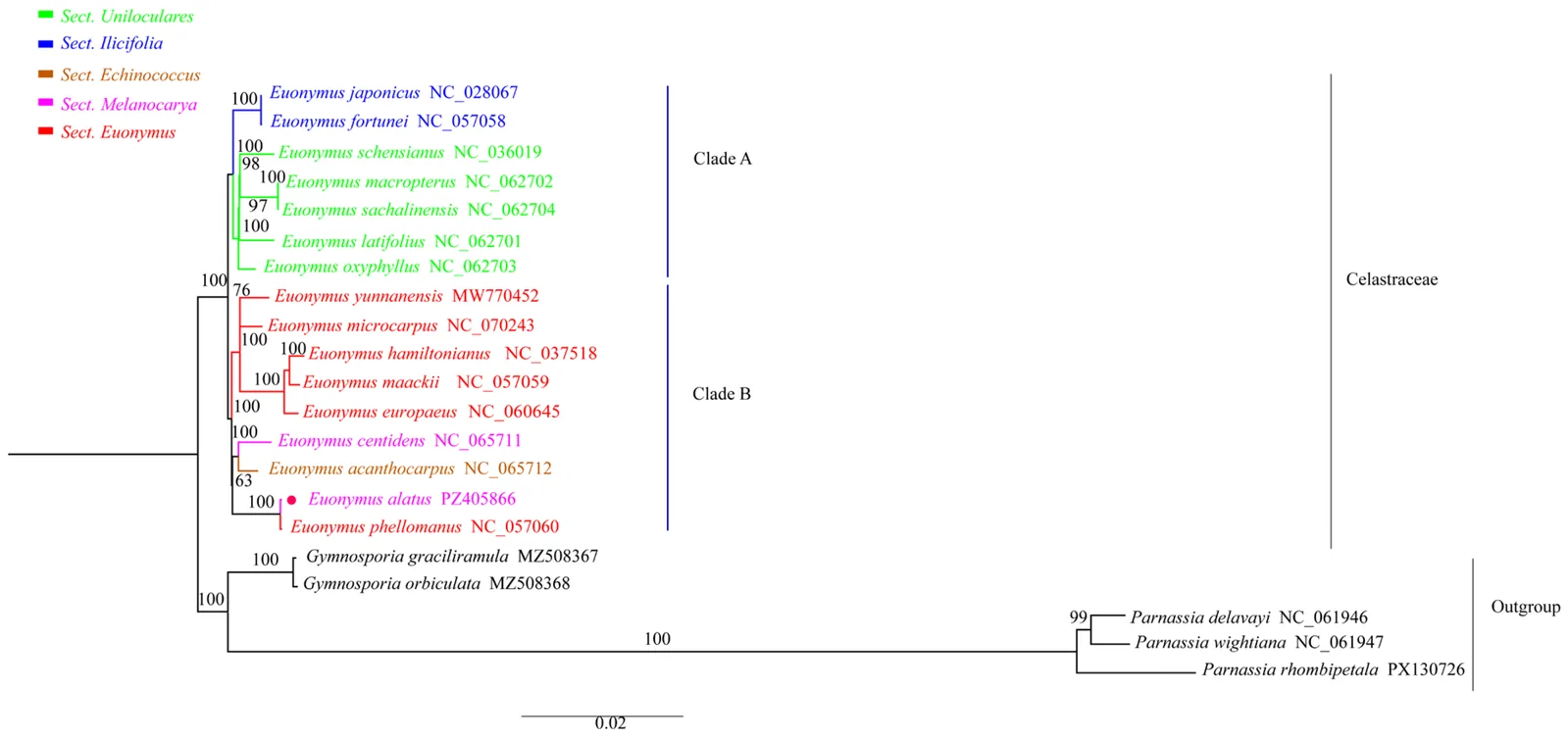
Phylogenetic tree of *E. alatus* and its closely related species constructed by the ML method.

Phylogenetic analysis revealed that the 16 *Euonymus* species formed a highly supported monophyletic group (ML-BS = 100%, BI-PP = 1.0) (Figure 6 and Figure S4), which was further divided into two major clades at the base: Clade A comprised seven species (*E. japonicus*, *E. fortunei*, *E. schensianus*, *E. macropterus*, *E. sachalinensis*, *E. latifolius*, and *E. oxyphyllus*), and Clade B comprised nine species (*E. yunnanensis*, *E. microcarpus*, *E. hamiltonianus*, *E. maackii*, *E. europaeus*, *E. centidens*, *E. acanthocarpus*, *E. alatus*, and *E. phellomanus*). The topologies of ML and BI trees were largely congruent, with all major nodes receiving high support values. *E. alatus* was most closely related to *E. phellomanus*.

## 4. Discussion

In this study, the complete chloroplast genome of *E. alatus* was assembled and annotated through high-throughput sequencing. Comparative genomics and phylogenetic analyses were subsequently conducted with 15 closely related *Euonymus* species, clarifying its structural features and phylogenetic position. The chloroplast genome of *E. alatus* is 157,416 bp in length, falling within the typical range of 120–160 kb, and exhibits a classical circular quadripartite structure. A total of 131 genes were annotated, and the gene composition and structural features are consistent with those of most terrestrial angiosperms [56]. The GC content in the IR regions is significantly higher than that in the LSC and SSC regions. The presence of the four rRNA genes within the IR regions is considered a major reason for this higher GC content [57].

IR contraction/expansion is a key driver of chloroplast genome evolution and size variation [58,59]. Our analysis of 16 *Euonymus* species revealed considerable IR/SC boundary variation. At LSC/IRb, *rps*19 position differed among species, indicating IR expansion in some cases, but exceptions occurred (e.g., IR expansion did not always reduce LSC length). Given our limited sampling, these boundary shifts may not be applicable to higher taxa. However, even minor displacements might affect gene integrity (e.g., *ycf1*) and could serve as genus-level markers. The apparent contradictions between IR expansion and LSC size suggest that internal LSC Indels may offset boundary effects. Hence, genome size variation likely reflects combined evolutionary forces rather than IR shifts alone.

The mVISTA and sliding window analyses showed that the IR region exhibited significantly lower variation than the LSC and SSC regions. Further, based on nucleotide polymorphism analysis, seven loci with the highest variation were identified in each of the coding and non-coding regions. These variable regions can serve as candidate markers for DNA barcoding and genetic diversity analysis. Among the variable regions identified, *ycf1*, *accD*, and *ndhF* stand out as having particularly high nucleotide diversity and proven utility in other plant groups [60,61,62,63,64,65,66,67,68]. Therefore, these three markers are recommended for priority consideration in subsequent analyses, while other promising regions (e.g., *rpl22*, *ccsA*, *rps15*, *rpl33*, and intergenic spacers) may also be retained for comparative evaluation depending on specific research objectives.

Repetitive sequences and SSRs are important components of the chloroplast genome, and their distribution characteristics are closely related to species evolution and molecular marker development [69,70,71,72]. The SSR loci identified in this study exhibited a strong A/T preference, a phenomenon thought to be associated with the base composition of the chloroplast genome [73]. Consistent with findings in most angiosperms [74,75,76,77,78], mononucleotide repeats accounted for the highest proportion of SSRs in the Euonymus alatus chloroplast genome, followed by dinucleotide, tetranucleotide, pentanucleotide, and trinucleotide repeats. This trend differs from that observed in some closely related species. For example, in *E. microcarpus*, the total number of SSRs was 282, with 199 mononucleotide, 10 dinucleotide, 82 tetranucleotide, 8 pentanucleotide, and 4 trinucleotide repeats [79]. In addition to species-specific differences, varying screening criteria may also affect the observed numbers. The SSRs identified in this study can serve as candidate markers for genetic diversity analysis and marker-assisted breeding of *Euonymus* species. However, their actual polymorphism requires experimental validation via primer design and population-level screening.

To evaluate selective constraints, we estimated the non-synonymous (dN) and synonymous (dS) substitution rates for all 74 chloroplast protein-coding genes using HyPhy v2.5, and calculated their ratio (ω = dN/dS). Based on ω values, we inferred purifying (ω < 1), neutral (ω = 1), or positive (ω > 1) selection acting on each gene. The complementary results from FUBAR and MEME indicated that, although the 16 chloroplast genomes from *Euonymus* species are generally highly conserved across all lineages (with no pervasive positive selection detected genome-wide), episodic positive selection signals can be detected on specific phylogenetic branches, suggesting a potential response to ecological or environmental pressures. This pattern is consistent with the general view that chloroplast genomes are functionally conserved yet locally variable [80]. Notably, the *clpP* gene exhibited an exceptionally strong signal of positive selection. This gene encodes the proteolytic subunit of the Clp protease complex, which plays a central role in chloroplast protein quality control. Positive selection on *clpP* has also been detected in other angiosperm lineages [81,82,83]. Given that *Euonymus* species are widely distributed across tropical and temperate regions worldwide [13,84], the strong positive selection signal suggests that *clpP* may play a potential role in the adaptation of *Euonymus* to diverse habitats. Future studies should focus on functional validation of *clpP* and its correlation with ecological factors to elucidate the specific mechanisms underlying its putative adaptive evolution. Furthermore, although previous studies identified *rpoB* as a putatively positively selected gene in *Euonymus* [85], our analysis did not confirm this finding; this discrepancy may be attributable to differences in species sampling and detection methods.

Studies have shown that unbalanced codon usage is an important mechanism for plants to adapt to environmental changes [86] and is widely observed across different types of organisms. The factors influencing codon usage bias are complex, involving base composition [87], protein structure [88], and gene expression levels [89], and are generally considered to be the result of mutation and selection [90]. This study analyzed the codon usage patterns and influencing factors of 53 CDSs from the chloroplast genome of *E. alatus*. The results indicate that the codon usage bias of this species is weak, with GC_3_ having a greater impact on codon usage bias. Based on the ENC-based theoretical assumption that genes with lower ENC values tend to be more highly expressed [91], a library of putatively high- and low-expression genes was established, and 17 optimal codons were identified in the *E. alatus* chloroplast genome. Except for UUG, all of these codons end with A or U, indicating a clear bias toward A/U at the third codon position. Similar patterns have been observed in other plants [92,93], consistent with the preference for A/U at the third codon position in dicotyledonous plants [94,95]. In this study, neutrality plot, ENC-plot, and PR2-plot analyses suggested that codon bias in the *E. alatus* chloroplast genome is influenced by both mutation pressure and natural selection, with selection appearing to play a relatively more prominent role under the mutation-selection balance framework. This pattern is comparable to findings in other plants [96,97,98], though the relative contribution of each force may vary across lineages. In addition, there are certain limitations in the codon usage bias analysis of this study. The optimal codon library was constructed using the top and bottom 10% cutoff thresholds, resulting in a relatively small library size of five genes for each extreme group, and the effective number of codons (ENC) serves only as an indirect proxy for gene expression level, which cannot completely replace direct expression data. Therefore, the above results of codon usage bias analysis only provide indicative trends, and the relevant conclusions require further validation in combination with transcriptomic expression data.

Phylogenetic analysis is a core method for investigating species relationships and taxonomic status. In this study, with five species from the genera *Gymnosporia* and *Parnassia* (*Celastraceae*) as outgroups, ML and BI trees were constructed based on complete chloroplast genome data. The results showed that the 16 *Euonymus* species were divided into two clades, which differ to some extent from morphological classification. For example, *E. alatus* is classified with *E. centidens* in the Flora of China, yet *E. alatus* shows a closer phylogenetic relationship with *E. phellomanus*, a species of Sect. *Euonymus*. Species of *Euonymus* are generally classified based on characteristics such as fruit morphology [99], flower number [100], style length [101], and aril [102]. However, the flowers of *Euonymus* species are inconspicuous and wither shortly after anthesis, complicating specimen identification [103]; therefore, species identification within this genus is challenging. Previous studies have employed combinations of nuclear and chloroplast DNA fragments to investigate the phylogeny of *Euonymus* and its allies, providing complementary evidence at different taxonomic levels. At the infrageneric level, Li et al., using broader taxon sampling, confirmed that among the five sections recognized in the Flora of China, only Sect. *Echinococcus* and Sect. *Kalonymus* correspond to the molecular groupings [22]. At the generic level, Simmons et al. found that *Glyptopetalum*, *Torralbasia*, and *Xylonymus* are all closely related to *Euonymus* sensu stricto, with their generic boundaries remaining questionable [21]. These results indicate that the classification of *Euonymus* remains uncertain at both infrageneric and generic levels. Currently, chloroplast genome data available for *Euonymus* in the NCBI database are limited, and chloroplast genomes reflect only maternal inheritance, which may not fully represent the true evolutionary relationships among species and may even conflict with nuclear gene-based phylogenies [104,105]. Future studies should include genomic data from more species and conduct comprehensive analyses incorporating nuclear genes and morphological characteristics to further refine the phylogenetic framework of the genus *Euonymus*.

## 5. Conclusions

This study analyzed the characteristics of the chloroplast genome of *E.alatus* and compared it with 15 other species within the same genus to construct a phylogenetic tree. The complete chloroplast genome of *E. alatus* is 157,416 bp in length and encodes a total of 131 genes. Genome structure validation confirmed its reliability through alignment with four previously published *Euonymus* chloroplast genomes, and our assembly provides the first fully annotated reference for this genus. A total of 67 tandem repeats, 49 interspersed repeats, and 40 simple sequence repeats (SSRs) were identified. No inversion or rearrangement events were observed among species of this genus. *E. alatus* exhibits a preference for codons ending in A/U, and its codon usage bias appears to be primarily shaped by natural selection based on the ENc-plot, neutrality plot, and PR2-plot analyses. Phylogenetically, *E. alatus* is most closely related to *E. phellomanus*. These results provide a reference for future studies on the phylogeny and evolution of the genus *Euonymus*. The identified SSRs and repeats may also facilitate marker development and taxonomic clarification in combination with nuclear data. Future comparative codon usage analyses incorporating more congeneric species, once their genomic data become available, would further distinguish lineage-specific patterns from general trends within *Euonymus*.

## Figures and Tables

**Table 1:** Annotation information of the chloroplast genomes of *E. alatus*.

Group of Genes	Name of Genes
Transfer Ribonucleic Acid (tRNA) (37)	*trnM-CAU*, *trnI-CAU(2)*, *trnL-CAA(2)*, *trnV-GAC(2)*, *trnE-UUC*, trnA-UGC *(2), *trnR-ACG(2)*, *trnN-GUU(2)*, *trnL-UAG*, trnI-GAU *(2), *trnP-UGG*, *trnW-CCA*, trnV-UAC *, *trnF-GAA*, trnL-UAA *, *trnT-UGU*, *trnS-GGA*, *trnG-GCC*, *trnS-UGA*, *trnT-GGU*, *trnE-UUC*, *trnY-GUA*, *trnD-GUC*, *trnC-GCA*, *trnR-UCU*, *trnS-GCU*, *trnQ-UUG*, trnK-UUU *, *trnH-GUG*, *trnfM-CAU*
Ribosomal Ribonucleic Acid (rRNA) (8)	*rrn16(2)*, *rrn23(2)*, *rrn4.5(2)*, *rrn5(2)*
Small subunit of ribosome (14)	*rps11*, rps12 *(2), *rps14*, *rps15*, *rps16*, *rps18*, *rps19*, *rps2*, *rps3*, *rps4*, *rps7(2)*, *rps8*
Large subunit of ribosome (11)	*rpl14*, rpl16 *, rpl2 *(2), *rpl20*, *rpl22*, *rpl23(2)*, *rpl32*, *rpl33*, *rpl36*
RNA polymerase (4)	*rpoA*, *rpoB*, rpoC1 *, *rpoC2*
NADH dehydrogenase (12)	NdhA *, ndhB *(2), *ndhC*, *ndhD*, *ndhE*, *ndhF*, *ndhG*, *ndhH*, *ndhI*, *ndhJ*, *ndhK*
Photosystem I (5)	*psaA*, *psaB*, *psaC*, *psaJ*, *psaI*
Photosystem II (15)	*psbA*, *psbB*, *psbC*, *psbD*, *psbE*, *psbF*, *psbH*, *psbI*, *psbJ*, *psbK*, *psbM*, *psbN*, *psbL*, *psbT*, *psbZ*
Cytochrome b/f complex (6)	*petA*, petB *, petD *, *petG*, *petL*, *petN*
ATP synthase (6)	*atpA*, *atpB*, *atpE*, atpF *, *atpH*, *atpI*
Large subunit of rubisco (1)	*rbcL*
Maturase (1)	*matK*
Protease (1)	ClpP **
Envelope membrane protein (1)	*cemA*
Subunit of acetyl-carboxylase (1)	*accD*
C-type cytochrome synthesis (1)	*ccsA*
Translation initiation factor (1)	*infA*
Proteins of unknown function (6)	*ycf1(2)*, *ycf2(2)*, ycf3 **, *ycf4*

Note: Number of copies of multi-copy genes; *: one intron; **: two introns.

**Table 2:** Characterization analysis of genes with introns in chloroplast genome of *E. alatus*.

Gene	Location	Exon (bp)			Intron (bp)	
		I	II	III	I	II
*atpF*	LSC	144	411		700	
*clpP*	LSC	71	292	231	857	629
*ndhA*	SSC	551	541		1223	
*ndhB*	IR	777	756		685	
*petB*	LSC	6	642		780	
*petD*	LSC	8	475		716	
*rpoC1*	LSC	432	1617		839	
*rpl16*	LSC	9	399		1164	
*rpl2*	IR	391	434		679	
rps12 *	LSC/IR	114	231	27	-	-
*trnA-UGC*	IR	37	36		799	
*trnI-GAU*	IR	37	35		951	
*trnV-UAC*	LSC	39	37		702	
*trnL-UAA*	LSC	35	50		533	
*trnK-UUU*	LSC	37	35		2502	
*ycf3*	LSC	126	226	155	731	722
*TrnG-UCC*	LSC	23	48		765	

Note: * indicates a trans-spliced gene; ‘-’ indicates intron absence; blank cells indicate no third exon.

**Table 3:** MEME analysis of episodic positive selection in chloroplast genes of *Euonymus*.

No.	Gene	Total Sites	Sites *p* < 0.05	Sites *p* < 0.01	Positive Selection
1	*clpP*	195	82	21	Yes
2	*psbM*	43	3	0	Yes
3	*petA*	309	2	0	Yes
4	*psaA*	687	2	0	Yes
5	*psbB*	467	2	0	Yes
6	*atpB*	463	1	0	Yes
7	*cemA*	207	1	1	Yes
8	*ndhA*	328	1	0	Yes
9	*ndhK*	215	1	0	Yes
10	*psbA*	325	1	0	Yes
11	*rps19*	88	1	0	Yes
12	*atpH*	77	0	0	No
13	*atpI*	233	0	0	No
14	*ndhC*	107	0	0	No
15	*ndhG*	163	0	0	No
16	*ndhH*	357	0	0	No
17	*ndhJ*	146	0	0	No
18	*petB*	196	0	0	No
19	*petD*	148	0	0	No
20	*psaC*	73	0	0	No
21	*psbC*	431	0	0	No
22	*psbE*	76	0	0	No
23	*psbK*	56	0	0	No
24	*psbT*	32	0	0	No
25	*psbZ*	57	0	0	No
26	*rpl14*	118	0	0	No
27	*rpl20*	107	0	0	No
28	*rpl23*	85	0	0	No
29	*rps11*	131	0	0	No
30	*rps16*	94	0	0	No
31	*rps18*	103	0	0	No
32	*rps4*	193	0	0	No
33	*rps7*	147	0	0	No
34	*rps8*	122	0	0	No
35	*ycf3*	157	0	0	No

**Table 4:** RSCU analysis of protein-coding region in the chloroplast of *E. alatus*.

No.	AA	Condon	Number	RSCU	No.	AA	Condon	Number	RSCU
1	Phe	UUU	782	1.32	31	Tyr	UAU	614	1.59
2		UUC	401	0.68	32		UAC	157	0.41
3	Leu	UUA	705	1.88	33	His	CAU	396	1.52
4		UUG	451	1.20	34		CAC	126	0.48
5		CUU	484	1.29	35	Gln	CAA	587	1.54
6		CUC	147	0.39	36		CAG	174	0.46
7		CUA	304	0.81	37	Asn	AAU	743	1.50
8		CUG	155	0.41	38		AAC	245	0.50
9	Ile	AUU	853	1.47	39	Lys	AAA	863	1.49
10		AUC	348	0.60	40		AAG	294	0.51
11		AUA	542	0.93	41	Asp	GAU	660	1.59
12	Met	AUG	471	1.00	42		GAC	172	0.41
13	Val	GUU	430	1.49	43	Glu	GAA	861	1.49
14		GUC	145	0.50	44		GAG	298	0.51
15		GUA	415	1.43	45	Cys	UGU	170	1.47
16		GUG	168	0.58	46		UGC	62	0.53
17	Ser	UCU	442	1.72	47	Arg	CGU	284	1.34
18		UCC	241	0.94	48		CGC	95	0.45
19		UCA	292	1.14	49		CGA	288	1.36
20		UCG	144	0.56	50		CGG	84	0.40
21		AGU	330	1.29	51	Arg	AGA	369	1.75
22		AGC	91	0.35	52		AGG	147	0.70
23	Pro	CCU	326	1.54	53	Gly	GGU	476	1.33
24		CCC	162	0.76	54		GGC	170	0.47
25		CCA	242	1.14	55		GGA	549	1.53
26		CCG	119	0.56	56		GGG	237	0.66
27	Thr	ACU	445	1.66	57	Ala	GCU	524	1.80
28		ACC	207	0.77	58		GCC	191	0.66
29		ACA	307	1.14	59		GCA	299	1.03
30		ACG	115	0.43	60		GCG	151	0.52

**Table 5:** Putative optimal codons in the chloroplast genome of *E. alatus*.

Amino Acid	Codon	Highly Expressed Gene		Low Expressed Gene		∆RSCU
		Number	RSCU	Number	RSCU	
Phe	**UUU** *	19	1.58	22	1.47	0.11
	UUC	5	0.42	8	0.53	−0.11
Leu	**UUA** *	24	1.97	26	1.88	0.09
	**UUG** ***	20	1.64	14	1.01	0.63
	**CUU** *	19	1.56	18	1.30	0.26
	CUC	1	0.08	8	0.58	−0.5
	CUA	7	0.58	11	0.80	−0.22
	CUG	2	0.16	6	0.43	−0.27
Ile	**AUU** *	27	1.56	35	1.36	0.2
	AUC	6	0.35	14	0.55	−0.2
	AUA	19	1.10	28	1.09	0.01
Met	AUG	18	1.00	22	1.00	0
Val	GUU	10	1.25	20	1.38	−0.13
	GUC	3	0.38	6	0.41	−0.03
	**GUA** **	16	2.00	23	1.59	0.41
	GUG	3	0.38	9	0.62	−0.24
Ser	UCU	10	1.54	18	1.69	−0.15
	UCC	5	0.77	10	0.94	−0.17
	UCA	7	1.08	11	1.03	0.05
	UCG **	5	0.77	4	0.38	0.39
	AGU	10	1.54	18	1.69	−0.15
	AGC	2	0.31	3	0.28	0.03
Pro	**CCU** *	15	1.71	18	1.50	0.21
	CCC	7	0.80	12	1.00	−0.2
	CCA	8	0.91	13	1.08	−0.17
	CCG *	5	0.57	5	0.42	0.15
Thr	**ACU** ***	15	1.71	12	1.04	0.67
	ACC	5	0.57	11	0.96	−0.39
	ACA	12	1.37	17	1.48	−0.11
	ACG	3	0.34	6	0.52	−0.18
Ala	**GCU** ***	17	1.79	17	1.06	0.73
	GCC	2	0.21	19	1.19	−0.98
	**GCA** **	13	1.37	17	1.06	0.31
	GCG	6	0.63	11	0.69	−0.06
Tyr	**UAU** ***	14	1.87	26	1.37	0.5
	UAC	1	0.13	12	0.63	−0.5
His	**CAU** *	11	1.83	15	1.58	0.25
	CAC	1	0.17	4	0.42	−0.25
Gln	CAA	18	1.80	23	1.77	0.03
	CAG	2	0.20	3	0.23	−0.03
Asn	AAU	20	1.54	33	1.69	−0.15
	AAC *	6	0.46	6	0.31	0.15
Lys	**AAA** **	36	1.71	33	1.40	0.31
	AAG	6	0.29	14	0.60	−0.31
Asp	GAU	7	1.00	31	1.55	−0.55
	GAC ***	7	1.00	9	0.45	0.55
Glu	**GAA** **	23	1.64	29	1.29	0.35
	GAG	5	0.36	16	0.71	−0.35
Cys	**UGU** ***	3	2.00	8	1.33	0.67
	UGC	0	0.00	4	0.67	−0.67
Arg	CGU	14	1.20	15	1.34	−0.14
	CGC	3	0.26	9	0.81	−0.55
	**CGA** ***	23	1.97	9	0.81	1.16
	CGG	3	0.26	4	0.36	−0.1
	AGA	20	1.71	21	1.88	−0.17
	AGG	7	0.60	9	0.81	−0.21
Gly	GGU	17	1.39	25	1.39	0
	GGC	4	0.33	9	0.50	−0.17
	**GGA** *	19	1.55	25	1.39	0.16
	GGG	9	0.73	13	0.72	0.01

Note: * Indicates ΔRSCU ≥ 0.08; ** indicates ΔRSCU ≥ 0.3; *** indicates ΔRSCU ≥ 0.5; Underlined ‘_’ indicates RSCU > 1; Bold type indicates that the codon is the optimal codon.

## Data Availability

The genome sequence data that support the findings of this study are available in GenBank under the accession PZ405866. The associated BioProject, SRA, and BioSample numbers are PRJNA1469901, SRR38813415, and SAMN60387182, respectively.

## Supplementary Materials

The following supporting information can be downloaded at https://www.mdpi.com/article/10.3390/cimb48080822/s1.
