# Supplementary material for: Comparative Chloroplast Genomics, Codon Usage Bias, and Phylogenetic Placement of Euonymus alatus
Source: Curr Issues Mol Biol. 2026 Aug 12;48(8):822. doi: 10.3390/cimb48080822 (PMC13510332; doi:10.3390/cimb48080822)
Supplement: Supplementary file 1 [file cimb-48-00822-s001.zip › supplementayry figures S1-S4.pdf]

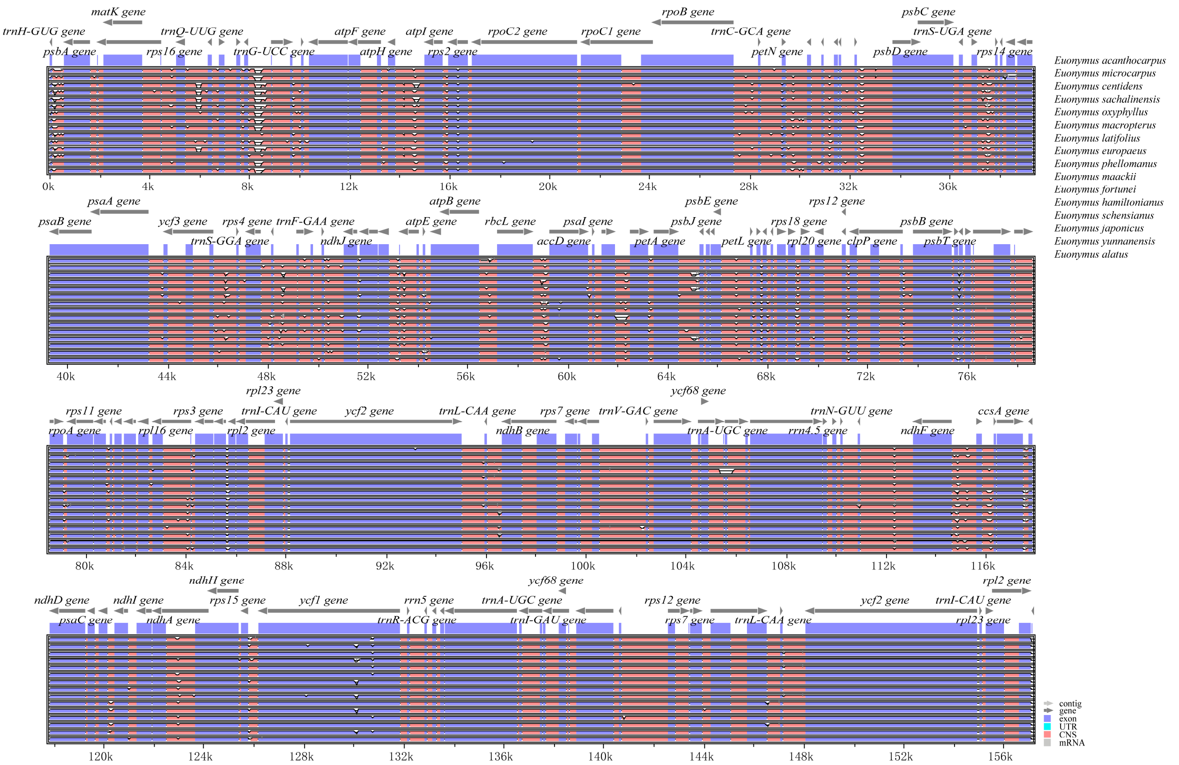

Supplementary Figure S1. Visualization for comparison of 16 Euonymus chloroplast genomes.

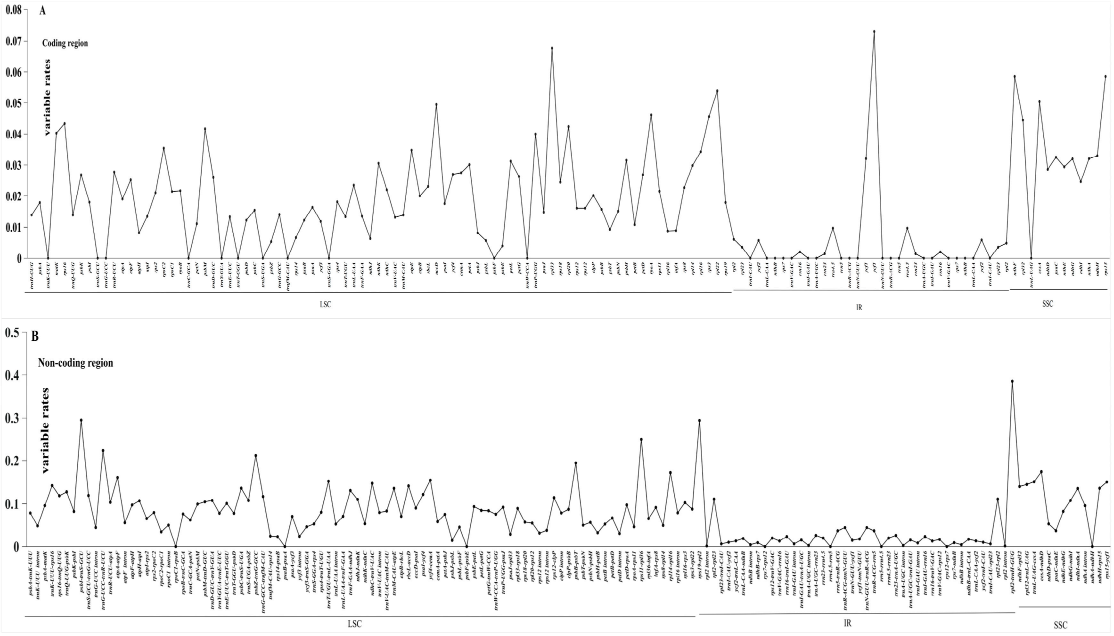

Supplementary Figure S2. Percentages of variable characters among chloroplast genomes of 16 Euonymus plant. (A) Coding region, (B) Noncoding regions.

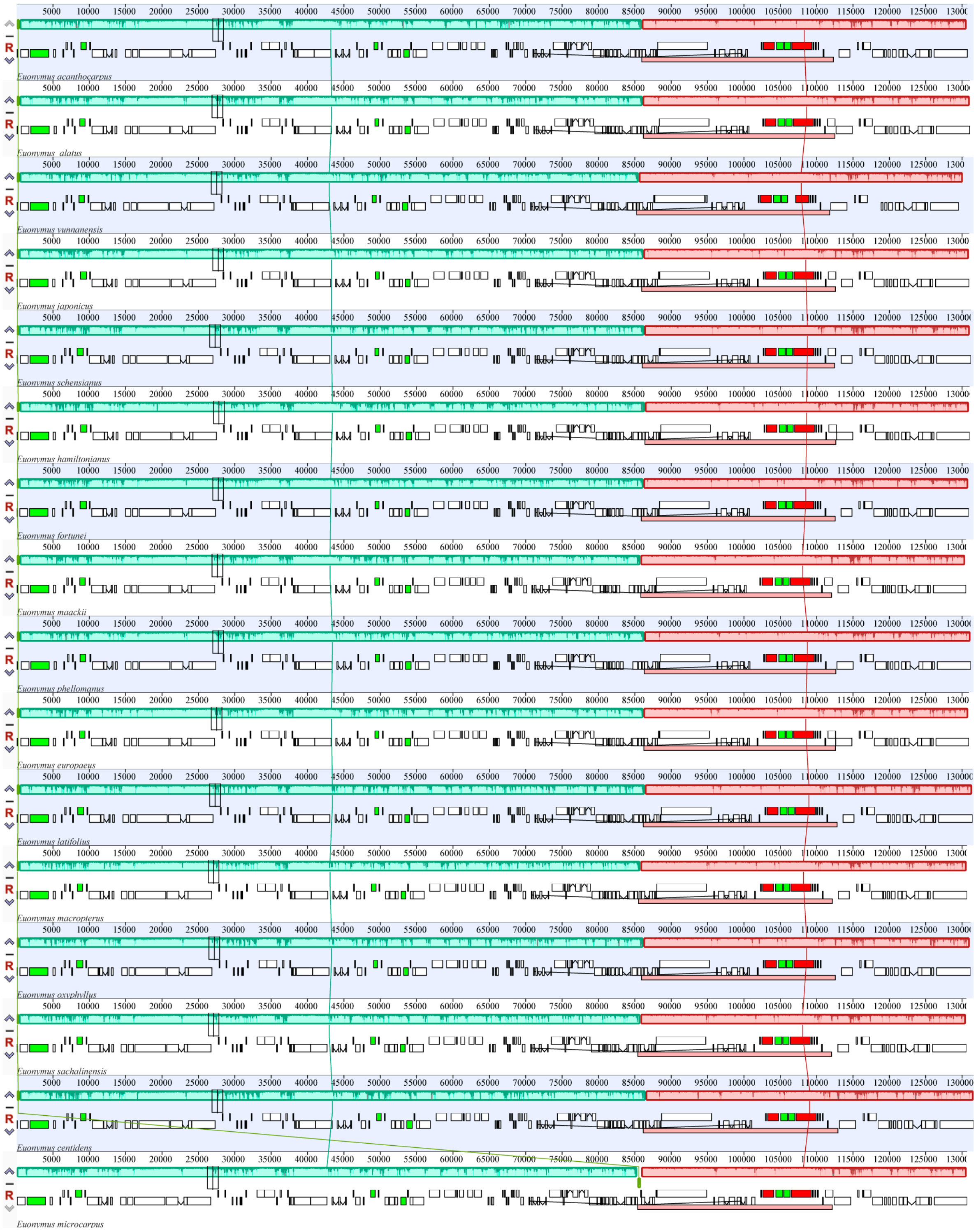

Supplementary Figure S3. Collonearity analysis of chloroplast genomes from16 species in Euonymus.

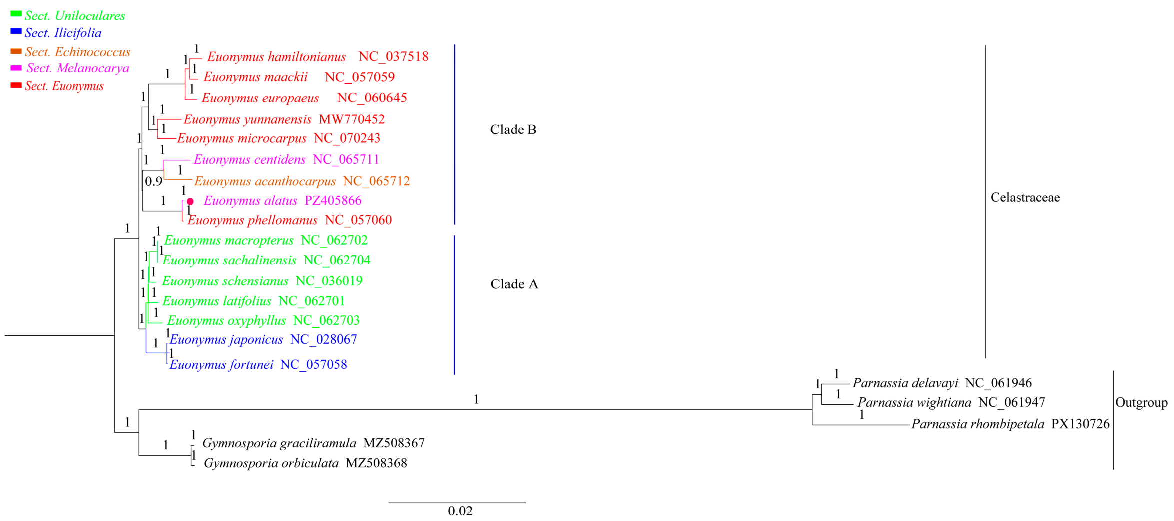

Supplementary Figure S4. Phylogenetic tree of E. alatus and its closely related species constructed by BI method.
